# Differential monocular vs. binocular pupil responses from melanopsin-based photoreception in patients with anterior ischemic optic neuropathy

**DOI:** 10.1038/srep10780

**Published:** 2015-06-15

**Authors:** Chrysanthi Tsika, Sylvain V. Crippa, Aki Kawasaki

**Affiliations:** 1 Department of Ophthalmology, University of Lausanne, Hôpital Ophtalmique Jules Gonin and Asile des Aveugles Foundation, Avenue de France 15, Lausanne, Switzerland 1004; 2 Department of Ophthalmology, Medical School of the University of Crete, Greece Eye Clinic, University Hospital of Heraklion, Crete Greece, 71013 Voutes Heraklion Crete, Greece.

## Abstract

We examined the effect of anterior ischemic optic neuropathy (AION) on the activity of intrinsically photosensitive retinal ganglion cells (ipRGCs) using the pupil as proxy. Eighteen patients with AION (10 unilateral, 8 bilateral) and 29 age-matched control subjects underwent chromatic pupillometry. Red and blue light stimuli increasing in 0.5 log steps were presented to each eye independently under conditions of dark and light adaptation. The recorded pupil contraction was plotted against stimulus intensity to generate scotopic and photopic response curves for assessment of synaptically-mediated ipRGC activity. Bright blue light stimuli presented monocularly and binocularly were used for melanopsin activation. The post-stimulus pupil size (PSPS) at the 6th second following stimulus offset was the marker of intrinsic ipRGC activity. Finally, questionnaires were administered to assess the influence of ipRGCs on sleep. The pupil response and PSPS to all monocularly-presented light stimuli were impaired in AION eyes, indicating ipRGC dysfunction. To binocular light stimulation, the PSPS of AION patients was similar to that of controls. There was no difference in the sleep habits of the two groups. Thus after ischemic injury to one or both optic nerves, the summated intrinsic ipRGC activity is preserved when both eyes receive adequate light exposure.

The intrinsically photosensitive retinal ganglion cells (ipRGCs) express the photopigment melanopsin which confers the capacity of intrinsic phototransduction^1^,^2^. The spectral sensitivity of melanopsin is broad with a peak sensitivity to short wavelength light in the blue portion of the visual spectrum. Compared to rod and cone photoreceptors, the melanopsin photoresponse is slow and insensitive^2^,^3^,^4^. Its unique features are, however, the ability to sustain a steady-state depolarization of ipRGCs during continuous illumination and to maintain ipRGC firing for some time after termination of the stimulus light^2^.

The ipRGCs mediate non-visual, light-dependent functions such as entrainment of the circadian clock and pupillary responses to light changes^3^,^5^. In macaque monkeys whose eyes have been pharmacologically treated to block hyperpolarization of rods and cones, the intrinsic melanopsin photoresponse can be isolated and it is capable of driving a strong pupillary contraction^6^. In eyes with intact rods and cones, the overlap of spectral and luminance sensitivities between rod, cone and melanopsin photosystems precludes isolation of the melanopsin photoresponse. In normal eyes, the light signal relayed by ipRGCs is an integration of their intrinsic, melanopsin mechanism and extrinsic, synaptically-transmitted influences from rods and cones^4^,^7^,^8^. The rapid kinetics of rod and cone activation are primarily reflected in the immediate pupil response to light onset and offset whereas the slow kinetics of the melanopsin photoresponse are better discerned in the post-stimulus pupillary behavior. Specifically, a protracted contraction of the pupil after termination of the light stimulus appears to be mediated primarily by melanopsin^6^,^7^,^8^,^9^,^10^,^11^,^12^.

The ipRGCs have a different morphology and retinal distribution compared to conventional vision-mediating retinal ganglion cells^4^,^5^. In addition, there is a growing body of evidence that ipRGCs display greater resistance to certain models of ganglion cell injury and death^13^,^14^,^15^,^16^,^17^,^18^,^19^. This robustness of ipRGCs is the basis for the visual-pupillary dissociation that is observed in patients with isolated hereditary mitochondrial optic neuropathy. These patients have significant visual loss and optic atrophy in both eyes and yet demonstrate normal pupil responses to light and have normally entrained sleep-wake cycles^19^,^20^,^21^.

On the other hand, glaucomatous optic neuropathy does not appear to spare ipRGCs and melanopsin-mediated functions. Two studies have shown that patients with advanced glaucoma have diminished pupil contraction amplitudes to light as well as a reduced post-illumination pupil contraction^22^,^23^. Glaucoma patients also show a reduction in the light-induced suppression of nocturnal melatonin secretion and have disturbances in sleep quality. Taken together, these findings implicate involvement of ipRGCs in glaucomatous optic neuropathy^24^,^25^.

Anterior ischemic optic neuropathy (AION) represents an optic nerve disorder in which the primary insult is insufficient perfusion to the optic nerve head. This leads to axoplasmic stasis and swelling, followed by axonal dropout and loss of retinal ganglion cells^26^. Presumably, the ischemic damage is non-selective and involves both visual retinal ganglion cells and ipRGCs. Clinical examination demonstrates loss of visual function and there may be impaired pupillary function that is detectable as a relative afferent pupillary defect (RAPD). The present study aimed to examine the effect of ischemic optic nerve damage on the functional integrity of ipRGCs through a more complete assessment of pupil responses to light. Specifically, we quantified the pupil response to light stimuli of varying spectral wavelength and intensity that were pre-selected to bias activation in favor of one photoreceptor system. The melanopsin photoresponse is specific to ipRGCs, and this was our primary parameter of interest. We assessed this intrinsic activity of ipRGCs from the post-stimulus pupil size following monocular as well as binocular light stimulation in patients with unilateral and bilateral AION. Finally, we surveyed the sleep habit of these patients via standardized questionnaires^27^,^28^,^29^ to understand how AION in one or both eyes might influence downstream physiologic processes such as sleep homeostasis which is strongly linked to melanopsin-based light signaling^30^.

## Results

The 18 patients with AION were 8 men and 10 women (aged 26 to 69 years) and the 29 control subjects were 10 men and 19 women (aged 24 to 70 years). Ten patients had unilateral AION of the non-arteritic type and henceforth are called the “unilateral group”. The “bilateral group” was comprised of one patient with bilateral non-arteritic AION and 7 patients with bilateral AION associated with optic disc drusen^31^.

The unilateral group was 4 men and 6 women having a median age of 67 years with a range between 50 to 69 years. The clinical characteristics of this group and a subgroup of 10 age-matched controls are given in Table 1. The affected eyes of the unilateral group demonstrated significantly reduced visual function (acuity, color vision and visual field) and significantly reduced thickness of the peripapillary retinal nerve fiber layer (RNFL). All affected eyes demonstrated an RAPD on clinical examination, though it was generally small (median 0.6 log-units, range 0.3 to 1.2 log-units). The RAPD was significantly correlated with the interocular difference in mean deviation (MD) of the visual field defect (correlation coefficient 0.64, p = 0.046) but not with visual acuity, color vision or RNFL. The ophthalmologic status of the unaffected eyes of the unilateral group was similar to age-matched control eyes.

**Figure 1:**
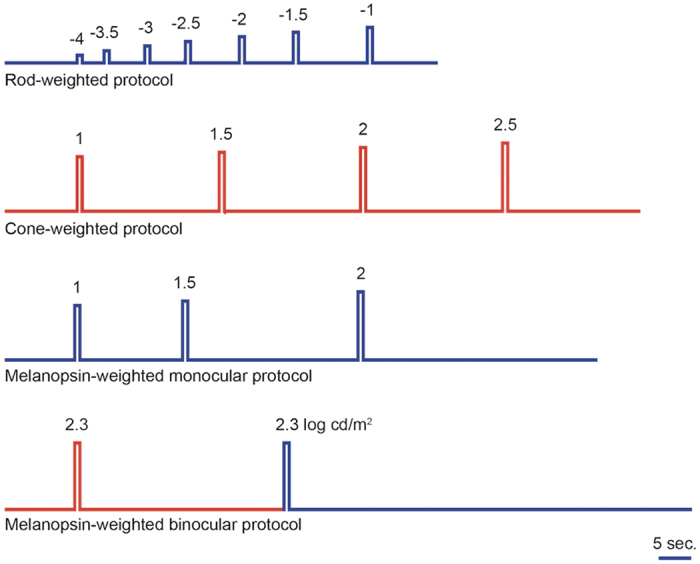
Schematic diagram of the light stimuli used in the four testing protocols. The rod-weighted protocol was presented to a dark adapted eye. The cone and melanopsin protocols were performed under light adapted conditions. The flat line indicates the time of darkness before and after each light stimulus. The vertical bar indicates the duration and timing of the light stimuli. The color of the bar indicates the color of the light stimulus. The number over each colored bar is the stimulus intensity in log cd/m2.

**Figure 2:**
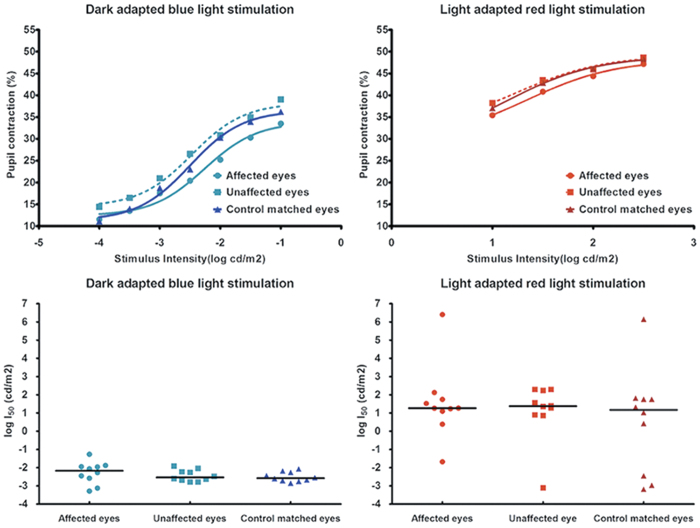
(Top) Pupil responses to blue light stimuli (rod-weighted protocol) and red light stimuli (cone-weighted protocol) for 10 patients with unilateral non-arteritic AION and 10 age-matched control subjects. An exponential curve is fitted through the median contraction amplitude obtained at each stimulus intensity for 3 groups of eyes: affected eyes of AION patients, unaffected eyes of AION patients and matched eyes of controls. (Bottom) Scatterplot of logI_50_ for affected eyes and unaffected eyes of 10 patients with unilateral AION and 10 age-matched control eyes. The horizontal bar represents the median value.

A schematic of the light stimuli used in 4 testing protocols is shown in Fig. 1. The response curve and the half-maximal response (logI_50_) derived from pupil responses to the rod-weighted protocol (dark-adapted, dim blue light stimuli) and to the cone-weighted protocol (light adapted, bright red light stimuli) are shown in Fig. 2 for the unilateral group and age-matched controls. Compared to unaffected eyes and control eyes, eyes with AION showed diminished pupil contraction amplitudes and generally less sensitive scotopic and photopic response curves. As expected, the logI_50_ for the rod-weighted protocol was higher in affected eyes compared to unaffected eyes and controls eyes but the difference was not significant (median logI_50_ = −2.17 log cd/m2 for affected eyes, −2.53 log cd/m2 for unaffected eyes, −2.58 log cd/m2 for controls, p = 0.37). For the cone-weighted protocol, there was no significant difference in the median logI_50_ between affected eyes, unaffected eyes and controls eyes (p = 0.83).

**Figure 3:**
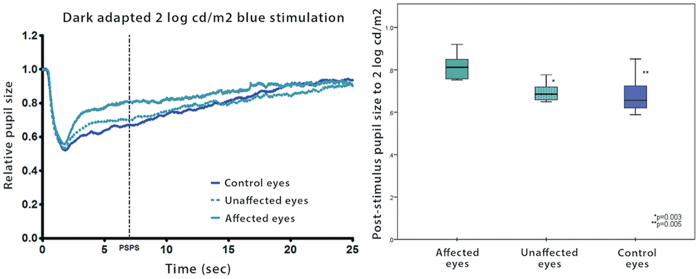
*Left:* Pupillometric recording of the pupil response (median) to a blue light stimulus (2.0 log cd/m2) from affected and unaffected eyes of 10 patients with unilateral non-arteritic AION and matched eyes from 10 age-matched control subjects. *Right*: Box plot of the PSPS (pupil size at the 6th second after light termination shown by a dotted line on the tracing) determined for the 2.0 log cd/m2 blue light stimulus for the same 3 groups of eyes. The center bar is the median value, the box limits represent the 25th and 75th percentiles and the bar limits are the 5th and 95th percentiles. P-values are given for comparison of affected eyes to unaffected eyes and affected eyes to control eyes.

As previously mentioned, the intrinsic melanopsin response to light is sluggish and for this reason, when an activating light is abruptly turned off, the pupil tends to stay contracted instead of re-dilating immediately^6^,^11^. We measured the post-stimulus pupil size (PSPS) as a marker of melanopsin activation to different light intensities in each eye independently. At intensities of 1.0 log cd/m2 and 1.5 log cd/m2, there was no difference in the median PSPS between affected eyes, unaffected eyes and age-matched control eyes (p = 0.17 and p = 0.26, respectively). This is consistent with a previous study in which these lower light intensities failed to elicit a measurable post-illumination pupil contraction^32^. At a stimulus intensity of 2.0 log cd/m2, there was a significant difference in the PSPS between groups (p = 0.001). The pupil response from eyes affected by AION demonstrated a relative inability to maintain a post-illumination pupillary contraction compared to unaffected eyes and control eyes (Fig. 3). To a 2.0 log cd/m2 blue light, the median PSPS of affected eyes was significantly larger compared to fellow unaffected eyes (81.2 and 68.5, respectively, p = 0.005) and larger compared to control eyes (81.2 and 62.8, respectively, p = 0.003). Between unaffected eyes and controls eyes, there was no significant difference in PSPS (p = 0.18).

The bilateral AION group was 4 men and 4 women with a median age of 50 years and ranging between 26 and 65 years. Table 2 provides the ophthalmologic data of each eye for these 8 patients and summary data for 29 control eyes. The eye with the RAPD was named the worse eye and showed significantly greater visual field loss compared to the better eye (median MD for worse eyes = 15.1dB , for better eyes = 6.2 dB, difference in MD = 8.9dB, p = 0.042). There was no significant difference in visual acuity or RNFL thickness between the worse eye and better eye (difference in acuity = 0.2, p = 0.168 and difference in RNFL = 6 μm, p = 0.160).

The degree of visual dysfunction and the thinning of RNFL from AION were similar between the unilateral group and the bilateral group. The visual field loss of affected eyes of the unilateral group (median MD 10.8 dB) was intermediate between the better and worse eyes of the bilateral group but these differences were not significant (p = 0.12 and p = 0.197, respectively). The visual acuity of the unilateral group was lower (median 0.8) compared to the bilateral group whose median acuity in each eye was 1.0; this difference was also not significant (p = 0.051). The median RNFL thickness was 72, 74 and 62 microns (μm) for affected eyes of the unilateral group, better eyes of the bilateral group and worse eyes of the bilateral group, respectively; p = 0.316.

**Figure 4:**
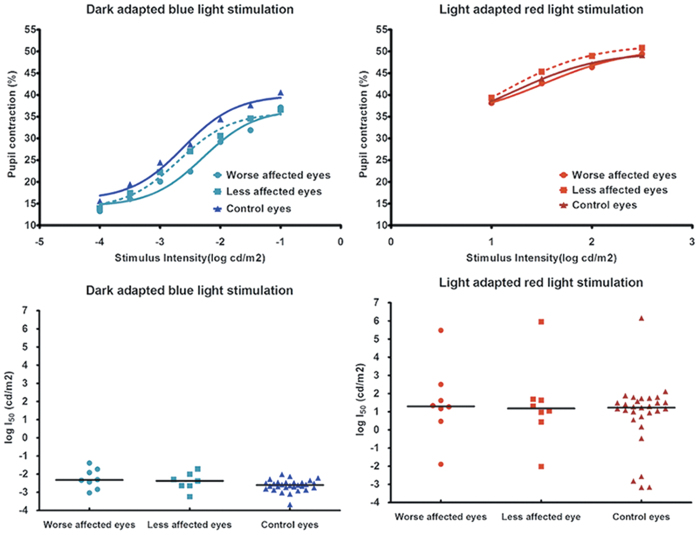
(Top) Pupil responses to blue and red light stimuli for 8 patients with bilateral AION and 29 control subjects. A curve is fitted through the median pupil contraction amplitude obtained at each stimulus intensity for 3 groups of eyes: the worse of two affected eyes of AION patients, the better eyes of AION patients and control eyes. (Bottom) Scatterplot of the logI_50_ for both affected eyes of 8 patients with bilateral AION and 29 control eyes. The horizontal bar represents the median value.

From the logI_50,_ the relative pupil deficit was also similar between the unilateral group and the bilateral group (p = 0.91 for cone-weighted protocol, p = 0.8 for rod-weighted protocol). The pupil responses from the rod-weighted protocol were reduced in both eyes of patients with bilateral AION (Fig. 4). As with unilateral AION, the rod-weighted logI_50_ for both affected eyes was higher compared to control eyes but the difference did not quite reach significance (median logI_50_ for better eyes = −2.40 log cd/m2, for worse eyes = −2.31 log cd/m2 compared to −2.60 log cd/m2 for control eyes, p = 0.10). The individual pupil responses to photopic red lights (cone-weighted protocol) were highly variable in all groups but the median response curves for these 3 groups of eyes were similar. There was no difference in the cone-weighted logI_50_ between groups (p = 0.76).

There was no significant difference in the PSPS between the two eyes of the bilateral group at any of the blue light intensities used in the monocular melanopsin-weighted protocol (1.0 log cd/m2 stimulus, p = 0.310; 1.5 log cd/m2 stimulus, p = 0.208; 2.0 log cd/m2 stimulus, p = 0.208). There was also no difference in the PSPS of either eye of the bilateral group as compared to affected eyes of the unilateral group (worse eyes, p = 0.79 and better eyes, p = 0.51).) The PSPS of the better eyes of the bilateral group tended to be smaller (more contracted) compared to control eyes but the difference was not significant at any intensity (p = 0.337, 0.507 and 0.077 for 1.0, 1.5 and 2.0 log cd/m2 stimulus intensities, respectively). There was, however, a significant difference in the post-illumination pupil response at the brightest intensity when comparing the PSPS of the worse eyes of the bilateral group to control eyes (0.81 and 0.63, respectively, p = 0.003).

**Figure 5:**
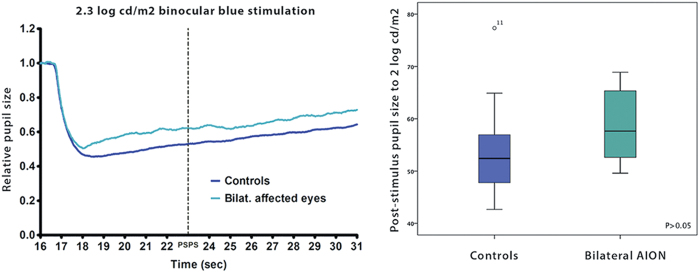
*Left:* Pupillometric recording of the pupil response (median) to a blue light stimulus (2.3 log cd/m2) presented binocularly to 8 patients with bilateral AION and 29 age-matched control subjects. The average of the pupil response of both eyes was used. Note that the pupil tends to stay contracted and re-dilation is very slow. The PSPS (pupil size at the 6th second after light termination) is shown by the dotted line. *Right*: Box plot of the average binocular PSPS for the two groups. The center bar is the median value, the box limits represent the 25th and 75th percentiles and the bar limits are the 5th and 95th percentiles. The difference in PSPS between patients with bilateral AION and controls is not statistically significant (p > 0.05).

From the binocular melanopsin-weighted protocol, the median PSPS to blue light at 2.3 log cd/m2 showed a significant group effect (p = 0.007) but this did not remain when groups were matched for age (Fig. 5). For the unilateral group and age-matched controls, the median PSPS was 0.61 and 0.54, respectively, and this difference was not significant (p = 0.20). Similarly for the bilateral group and 29 controls having similar median age, there was no difference in median PSPS (0.58 and 0.52 respectively, p = 0.051). We also determined the difference in PSPS between the blue light and an equivalent red light, as the red light is used as a reference light stimulus for absence of melanopsin contribution. The difference value was 0.27, 0.30 and 0.32 for the unilateral group, bilateral group and controls respectively, and these median values were not significantly different (p = 0.07).

For sleep habits, the unilateral group scored 70 on the Horne and Östberg (HO) Morningness-Eveningness questionnaire, as compared to 57 for the bilateral group and 60 for 29 controls. The controls and unilateral group were generally classified as morning types whereas the bilateral group was an intermediate type between morning and evening. From the Pittsburgh Sleep Quality Index (PSQI) questionnaire, 4 patients (2 unilateral AION, 2 bilateral AION) and 4 controls had a global score >5 which is the threshold score indicating poor sleep quality. The median scores for unilateral, bilateral and control groups were 3, 4 and 4 respectively, and the differences were not significant (p = 0.27).

## Discussion

Our patients with bilateral AION were generally younger that those with unilateral AION. This is consistent with other studies in which patients with drusen-associated AION are typically younger than patients with garden variety NAION^31^,^33^. All AION eyes demonstrated loss of visual field and reduction of the peripapillary RNFL compared to control eyes, and there were no significant differences in the visual function or RNFL between non-arteritic AION eyes and eyes with drusen-associated AION. The permanent visual deficit and optic atrophy are consistent with the pathological evidence of retinal ganglion cell apoptosis and death in eyes with AION^34^.

The clinical examination also revealed an RAPD which correlated with the visual field deficit, suggesting that ipRGCs are also affected by this disorder. However, the nature of AION-induced injury to ipRGCs is not known. As indicated by histopathologic studies in animals and humans in other optic neuropathies, ipRGCs may have greater resistance to injury and death compared to other vision-mediating retinal ganglion cells^13^,^14^,^16^,^17^,^18^,^19^. In this study, we assessed the intrinsic melanopsin photoresponse of ipRGCs as a measure of their functional integrity. Compared to controls, eyes with AION were less able to keep the pupil contracted following offset of a bright (2.0 log cd/m2) blue light, indicating less melanopsin influence on the post-illumination behavior of the pupil. In addition, AION eyes showed a significant reduction in the pupil responses to scotopic and photopic light stimuli, indicating that the pupil contraction driven largely by rods and cones is also impaired in AION. This latter finding raises the question whether trans-synaptic degeneration of outer retinal cells could be the basis for explaining all the pupil findings in this study. This is not likely as AION is a primary ischemic injury to the prelaminar portion of the optic nerve head. Following acute AION, there is axonal thinning and ganglion cell loss^34^. Animal models of AION indicate that the degenerative changes are limited to the inner retina and outer photoreceptors are not involved^35^,^36^. In humans, there is no definitive clinical evidence of outer retinal dysfunction.

Additionally, we have previously shown that the post-illumination pupil response is actually enhanced, and not diminished, in patients with primary outer photoreceptor degenerative disorders^37^. This is presumably from a pathologic alteration in ipRGC signal integration in which melanopsin input becomes increasingly dominant as rods and cones degenerate. Thus we believe the combination of visual deficit, optic atrophy, diminished pupil contraction and impaired post-illumination response in eyes with AION is best explained by non-selective ganglion cell death in this disorder.

Our finding of impaired post-illumination contraction following monocular bright blue light stimulation in eyes with AION is at odds with previous studies which found no difference in this pupil response^32^,^37^,^38^. Small sample size and methodologic variety can probably explain this discrepancy. Each study used a different stimulus light under different states of retinal light adaptation to activate melanopsin and each selected a different aspect of the post-stimulus pupillary dynamics as the measure of melanopsin activity. Our current testing protocol is an update from previous versions of chromatic pupillometry that we have used and has been optimized for preferential activation of one photoreceptive system^32^,^39^. Protocols using the late phase of the post-stimulus response are prone to excessive variability as supranuclear influences on pupillary re-dilation are known to increase with increasing time after light termination^40^.

In this study, patients with bilateral AION had visual dysfunction and loss of RNFL that was similar between the two affected eyes. When tested independently, each eye also demonstrated diminished pupil responses to all light stimuli. When the melanopsin-weighted light stimuli were presented to both eyes simultaneously, these patients with bilateral AION showed no difference in the post-stimulus pupil response compared to that of patients with unilateral AION and of controls. Reasons for the preservation of summated intrinsic activity of ipRGCs to binocular but not monocular stimulation are not known and herein are purely speculative. One possibility is binocular summation. Visual tasks based on binocular viewing are superior to monocular vision of either eye alone. The model for binocular summation is complex, occurs in multiple stages that include the retina, thalamus and cortex, and involves mechanisms of gain control and reciprocal inhibition^41^,^42^. Like conventional retinal ganglion cells, ipRGCs are integrated into the retinal circuitry, so it may be that this phenomenon also applies to non-visual, light-mediated functions such as the pupil response. A related notion is a central threshold of activity. Due to hemi-decussation of axons at the optic chiasm, the ipRGC signals from one eye synapse at the ipsilateral and the contralateral olivary pretectal nucleus^43^. The pretectal olivary nucleus is the central integrator of all afferent pupillary input and it signals pupillomotor output to the Edinger-Westphal nuclei. Binocular pupillary input, even from two damaged optic nerves, may be sufficient to reach a threshold level of activity at the olivary pretectal nuclei for sustaining pupillary contraction after stimulus offset. Further studies are needed to understand the effects of melanopsin-mediated light signaling from one eye compared to two eyes.

Finally, we noted with interest that sleep quality and sleep timing in our patients with bilateral optic nerve damage were not different compared to the 29 subjects in the control group. All were morning or intermediate types. All except 2 patients and 4 control subjects self-rated their sleep quality as good. The unilateral group was classified as a “definitely” morning type, but this qualification might be an effect of their older age. It is known that people tend to become more morning type with increasing age after 50 years^44^. We are cautious in drawing any conclusions from the sleep questionnaires as those used in this study may not be valid for detecting the kinds of sleep disorder associated with loss of ipRGC signaling of the hypothalamus. Also, if changes in sleep behavior had occurred at the onset of AION, it is possible that adaptation over time has rendered them subjectively unperceived at the present time.

In conclusion, AION results in functional damage to ipRGCs. In affected eyes, the pupil response mediated by either primarily synaptic or primarily intrinsic activation of ipRGCs was reduced. Unexpectedly, we found that the summated intrinsic response of ipRGCs was preserved if binocular light exposure was given. This was true for patients with unilateral and bilateral AION. Further studies with more rigorous methodologies on a wider range of optic nerve damage are needed to better define the impairment of ipRGC activity in patients with optic nerve disorders and to better understand how such impairment might affect other melanopsin-influenced physiologic functions such as sleep.

## Methods

### Subjects

All participating subjects gave written informed consent for study participation. The study was conducted at the Hôpital Ophtalmique Jules Gonin, Lausanne, Switzerland and conformed to the tenets of the Declaration of Helsinki. The study (protocol number 101-09) was reviewed and approved by the Commission d’Ethique de la Recherche sur l’être humain which is the local institutional committee for ethical research using human subjects for the canton of Vaud in Switzerland. Patients who had been diagnosed with AION and followed for at least 2 years in the neuro-ophthalmology unit of the Hôpital Ophtalmique Jules Gonin were identified from chart records. Patients with systemic inflammatory disease known to cause optic neuropathy such as giant cell arteritis, systemic cancer, diabetes mellitus, obstructive sleep apnea, major depression, retinal disorder and glaucoma were excluded. In addition, the use of somnolent medication such as anti-depressives, anti-histaminics or anxiolytics and recent travel across two time zones in the preceding 3 months were exclusionary criteria because these factors influence the natural sleep habit. Because of the stringent exclusionary criteria, we identified only 50 eligible patients from a review of 200 charts classified as “AION”. Eighteen patients with chronic stable visual loss for the past 12 months were recruited to participate in the study (Table 1). Twenty nine healthy adults without past or present ocular disease participated voluntarily as controls for the study. The same exclusionary criteria applied to the controls. None of the patients or controls was using a topical or systemic medication which could adversely influence efferent pupil movements.

All participating subjects underwent ophthalmologic testing (C.T.) which included refraction for best-corrected acuity, Ishihara color vision test, threshold automated perimetry (Octopus 101, program G1 dynamic, Interzeag, Bern-Köniz, Switzerland), alternating light test with neutral density filters to look for a relative afferent pupillary defect (RAPD) and non-dilated funduscopy. Optical coherence tomography (OCT; Stratus 3000, Carl Zeiss, Meditec, Inc., Dublin, CA) was used to examine the macular structure and to quantify the peripapillary retinal nerve fiber layer (RNFL). Control subjects were required to have an acuity of 1.0 or better, normal color vision and normal perimetry with no RAPD and no macular lesion identified by funduscopy or OCT.

All subjects completed two sleep questionnaires and pupil testing as described in the following sections.

### Sleep questionnaires

The Horne & Ostberg Morningness-Eveningness questionnaire (HO) and the Pittsburgh Sleep Quality Index (PSQI) were used to assess sleep quality and habits. The HO questionnaire consists of 19 multiple-choice items referring to habitual rising and bed times, preferred times of mental and physical performance, and subjective alertness after rising and before going to bed^27^. The total numeric score ranges from 16 to 86 with higher scores indicating a greater preference for morning activities and earlier bedtime. Subjects are thus classified by their HO score: a score of 70–86 is ‘definitely morning type’, 59–69 is ‘moderately morning type’, 42–58 is ‘intermediate type’, 31–41 is ‘moderately evening type’ and 16–30 is ‘definitely evening type’^28^.

The PSQI provides a standardized quantitative measure of sleep quality^29^. It consists of 19 self-rated questions which are combined to assess seven components related to sleep latency, sleep duration, subjective sleep quality, sleep disturbances, habitual sleep efficiency, daytime dysfunction and use of sleep medications. Each component is rated from 0 (no difficulty) to 3 (severe difficulty). The global PSQI score ranges from 0 to 21 and a score >5 indicates poor sleep quality.

### Pupillometry

A ColorDome Ganzfeld bowl (Diagnosys, Lowell, Massachusetts) was used to present computer-controlled, wide-angle light stimuli of a specified duration at two predetermined spectral bandwidths, 635 ± 20 nm (red light) and 464 ± 26 nm (blue light). A chinrest set at the bowl opening gives a viewing angle of 45 degrees horizontal radius. The pupil diameter was monitored at 60 Hz and continuously recorded using a portable dual channel pupillometer (Arrington Research, Scottsdale, Arizona).

Computerized chromatic pupillography was performed under conditions of dark- and light-adaptation. Based on previous investigations using this apparatus^20^,^39^,^45^,^46^, we defined 4 testing protocols which selected for afferent pupillomotor input derived primarily from synaptic signals from outer retinal photoreceptors (rods and cones) or from its intrinsic melanopsin activity of ipRGCs . For the first three testing protocols, each eye was tested monocularly. The eye receiving light stimulation was also the recorded eye while the non-tested eye was kept under patch occlusion. By convention, the right eye was always tested first. For the fourth testing protocol, light stimulation was presented to both eyes simultaneously. The following paragraph summarizes the stimulus conditions of the 4 testing protocols (Fig. 1).

Test 1 (rod-weighted protocol): The tested eye was dark-adapted (0 cd/m2) for 10 minutes. The pupil of the dark-adapted eye was recorded for 10 seconds in total darkness and then in response to a series of dim, 1-second blue light stimuli starting at −4.0 log cd/m2 and increasing by 0.5 log-unit steps to –1.0 log cd/m2 (0 log cd/m2 = 1 cd/m2). The dark interval between light stimuli had been previously determined to allow the pupil to return to baseline size before the next light stimulation. Test 2 (cone-weighted protocol): The tested eye was light-adapted (90 cd/m2) for 5 minutes and the pupil was similarly recorded, as in the rod protocol, to 1-second red light stimuli ranging from 1.0 to 2.5 log cd/m2 (in 0.5 log steps). Test 3 (monocular melanopsin-weighted protocol) followed the cone-weighted protocol and thus was performed under photopic conditions. The light stimulation consisted of three 1-second blue lights at intensities of 1.0, 1.5 and 2.0 log cd/m2. After the last blue light stimulus, the pupil was recorded continuously in darkness for 35 seconds in order to observe the dynamics of the post-stimulus pupillary response^39^. Test 4 (binocular melanopsin-weighted protocol) was also performed under photopic conditions. A bright 1-second red light and then a bright 1-second blue light were presented. The red and blue lights were equiluminant for photopic sensitivity at 2.3 log cd/m2, according to the manufacturer of the Ganzfield apparatus (=14.9 log quanta for blue light and 15.1 log quanta for red light). The pupils were recorded in darkness for 30 seconds after the red light and for 60 seconds after the blue light.

### Analysis of pupil tracings. 

The video signal from the pupillometer was relayed to a processing board which recorded the maximal pupil diameter in real time into a text file. Analysis of the pupil data was performed in a spreadsheet. A customized filter was applied to remove gross artifacts from blinking and eye movements (Microsoft Excel 2010, Visual Basic for Applications). Baseline pupil size was defined from the average size during 1 second before onset of each light stimulus. Actual pupil size at any given time point was divided by baseline pupil size to convert all values to relative pupil size (RPS). For the rod-weighted and cone-weighted protocols, the immediate pupil contraction to light stimulation was determined as a maximal percentage change from baseline by the equation: (1 - minimum RPS) X 100. The calculated pupil contraction was plotted as a function of stimulus light intensity for each eye of each subject using nonlinear curve fit analysis in order to generate a stimulus response curve which characterized the retinal sensitivity to light over the intensity range of each protocol^45^. As the response curves appeared to reach an asymptote (maximal response), the light intensity at which a 50% pupil response occurred (logI_50_) was determined.

When melanopsin contribution to the pupil response is dominant, the pupil tends to stay small and contracted, instead of promptly re-dilating after the light stimulus has been turned off^2^,^4^,^11^. The degree of post-illumination pupillary contraction varies with the light stimulus characteristics. The outcome parameter we chose to estimate the post-stimulus contraction was the pupil size at the 6th second after light termination, or post-stimulus pupil size (PSPS). The post-stimulus pupil size was determined as the mean RPS between 5.5 s and 6.5 s after light termination. The PSPS as a clinical measure of melanopsin activity has been described and used in previous investigations^39^,^46^. In the binocular melanopsin-weighted protocol, a single blue light (2.3 log cd/m2 = 14.9 log quanta) was used as the melanopsin stimulus and an equiluminant red light stimulus was used as a reference stimulus . Afferent pupillary input derived from red light has little, if any, melanopsin contribution under conditions of this study^39^. Thus, for the binocular protocol, we calculated both the absolute blue light PSPS as well as the difference in the PSPS between red and blue light. In this way, potential confounding central effects on the post-stimulus pupil response could be detected and controlled.

### Statistical Analysis. 

Statistical analyses were carried out using the SPSS 21 software. Descriptive statistics were performed to characterize the distribution of age, acuity, color vision, MD, RNFL, RAPD, logI_50_ for rod-weighted and cone-weighted protocols, PSPS of melanopsin-weighted protocols and sleep questionnaire scores on three groups: controls, patients with unilateral AION and patients with bilateral AION. The data are presented as median values and the ranges. Due to the small number of subjects in each group, a non-normal distribution was assumed and non-parametric tests were applied for the comparisons. The Mann-Whitney U test was used for two independent samples, and for more than two independent samples, the Kruskal-Wallis test was used. The Wilcoxon signed rank test was used for paired samples comparisons. To look for a correlation between variables, a Spearman’s rank correlation coefficient was estimated. In all statistical analyses, two-sided tests were used and the results were considered statistically significant if p was less than 0.05.

## Additional Information

**How to cite this article**: Tsika, C. *et al.* Differential monocular vs. binocular pupil responses from melanopsin-based photoreception in patients with anterior ischemic optic neuropathy. *Sci. Rep.* **5**, 10780; doi: 10.1038/srep10780 (2015).

## Figures and Tables

**Table 1:** Clinical summary of 10 patients (P1-P10) with unilateral non-arteritic AION.

	**Age (in years)**	**Affected Eye**	**Visual Acuity (unaffected eye/affected eye) (decimal)**	**Mean defect (unaffected eye/affected eye) (dB)**	**RAPD (log-units)**	**Peripapillary RNFL (unaffected eye/affected eye (microns)**
P1	67	OD	1.0/0.4	0/15.8	1.2	90/78
P2	67	OD	1.0/0.8	−1.3/10.2	1.2	102/77
P3	66	OD	1.0/0.5	−0.8/22.5	0.6	92/52
P4	50	OS	1.0/1.0	0.6/13.7	0.3	91/56
P5	69	OD	1.0/1.0	−0.7/1.2	0.3	100/88
P6	68	OS	1.0/0.8	1.5/4.1	0.3	89/84
P7	66	OS	1.0/0.5	−0.4/3.3	0.6	103/67
P8	62	OD	1.0/0.8	1.0/5.2	0.3	101/84
P9	60	OS	1.0/0.1	1.0/15.2	0.9	88/45
P10	68	OD	1.0/0.8	−0.4/16.3	0.9	96/53
Affected eye of patients	Median: 67 (range: 50–69)		0.8 (0.1–1.0)	11.95 (1.2 to 22.5)	0.6 (0.3-1.2)	72 (45–88)
Unaffected eye of patients	–		a1.0 p = 0.002	a−0.2 (−1.3 to 1.5) p = 0.000210	0.0	a94 (88–103) p = 0.000181
Control eye of age-matched subjects (n = 10)	Median: 66 (range: 47–70)		b1.0 p = 0.002	b0.0 (−8.40 to 1.40) p = 0.000245	0.0	b85 (74–110) p = 0.01

The abbreviations included are the following: dB: decibel units, RAPD: relative afferent pupillary defect, RNFL: retinal nerve fiber layer. Statistically significant differences are depicted with small Latin letters as follows:

^a^significant difference (p < 0.05) between affected eyes and unaffected eyes of patients with unilateral non-arteritic AION.

^b^significant difference (p < 0.05) between affected eyes of patients and matched eyes of control subjects.

**Table 2:** Clinical summary of 8 patients with bilateral AION.

**Patient**	**Age (in years)**	**Visual Acuity (decimal) OD/OS**	**Mean defect (dB) OD/OS**	**RAPD (log-units)**	**Peripapillary RNFL (microns) OD/OS**
P1	65	1.0/0.4	13.3/19.4	0.9 OS	56/57
P2	47	1.0/1.0	8.6/13	0.3 OS	67/63
P3	26	0.8/1.0	21.3/0.6	0.9 OD	49/100
P4	36	1.0/1.0	4/13.7	0.9 OS	105/53
P5	29	1.0/1.0	5.2/16.7	0.3 OS	71/66
P6	53	1.0/1.0	10/16	0.6 OS	57/59
P7	53	1.0/1.0	5/11.7	0.3 OS	55/53
P8	62	1.0/1.0	2.9/9.1	0.3 OS	77/81
All patients (n = 8)	Median: 50 (range 26–65)	1.0(0.8–1.0)/1.0(0.4–1.0)	6.9(2.9–21.3)/13.35(0.6–19.4)	0.5 (0.3-0.9)	62(49–105)/61(53–100)
Control eye of healthy subjects (n = 29)	Median: 46 (range 24–70)	1.0/1.0	1.66(−6.9 –2.3)/1.96(−8.4–1.4)	0.0	92(75–110)/92(74–111)

The abbreviations included are the following: dB: decibel units, RAPD: relative afferent pupillary defect, RNFL: retinal nerve fiber layer.
